# Effect of Step Load Based on Time under Tension in Hypoxia on the ACL Pre-Operative Rehabilitation and Hormone Levels: A Case Study

**DOI:** 10.3390/jcm13102792

**Published:** 2024-05-09

**Authors:** Joanna Motowidło, Katarzyna Stronska-Garbien, Marta Bichowska-Pawęska, Maciej Kostrzewa, Adam Zając, Krzysztof Ficek, Miłosz Drozd

**Affiliations:** 1 Department of Sports Training, Institute of Sport Sciences, The Jerzy Kukuczka Academy of Physical Education, 40-065 Katowice, Poland; k.stronska@awf.katowice.pl (K.S.-G.); m.kostrzewa@awf.katowice.pl (M.K.); a.zajac@awf.katowice.pl (A.Z.); krzysztof.ficek@galen.pl (K.F.); 2 Faculty of Physical Education, Gdansk University of Physical Education and Sport, 80-336 Gdansk, Poland; marta.bichowska@awf.gda.pl

**Keywords:** injury prevention, time under tension, tempo, periodization, hypoxia, preoperative rehabilitation, step load, VO_2_ max, isokinetic, ACL

## Abstract

The aim of the study was to determine the effect of step load in hypoxia on the effectiveness of preoperative rehabilitation (PR) and hormone levels based on a case study. **Introduction**: We assessed the impact of variables such as rate of movement and time under tension (TUT) in normobaric hypoxia on the levels of growth hormone (GH), insulin-like growth factor 1 (IGF-1), and erythropoietin (EPO). Additionally, the impact of step load on the hypertrophy and strength of knee extensors and flexors was assessed. **Methods**: The work uses a case study, the research subject of which was a 23-year-old female professional handball player. The tests included an isokinetic assessment of the peak torque of knee extensors and flexors as well as body composition analysis. **Results**: The results showed a more than (10.81-fold) increase in GH after the microcycle with time under tension (TUT). The deficit between the lower limbs was also reduced. **Conclusions**: Using a hypoxic environment based on an appropriate altitude, combined with changes such as a short rest break between sets and a controlled tempo of movement with an eccentric phase, TUT may offer an alternative to the PR process, especially among athletes who care about fast RTS.

## 1. Introduction

Handball is one of the most popular team disciplines in Europe, characterized by changes in direction, acceleration, and sudden braking, which is related to technical and tactical requirements. The ability to carry out these activities at short intervals results in the accumulation of load on the musculoskeletal system. The frequency of injuries is 108 per 1000 h of play, which allows us to conclude that an injury occurs every 9.2 championship matches on average. The most common injuries in handball are on the lower limbs (42%), followed by the head (23%), on the upper limbs (18%), and on the trunk (14%). Most injuries occur as a result of contact with another player. In particular, the knee joint is subject to mechanical stress. Dynamic situations have been confirmed to be the most dangerous for the knee joint, especially the anterior cruciate ligament (ACL) [1].

Depending on the level of sport practised, the risk of anterior cruciate ligament damage in women is 1.6 to 4.6 times higher than in men. The factors of damage to the anterior cruciate ligament can be divided into external, contact, and internal, non-contact. In recent years, the risk of injury has increased significantly. It was suggested that women’s injuries can be influenced by internal factors such as anatomical and hormonal differences, lack of strength or coordination, and previous injuries, as well as external factors such as the type of shoe or the pitch surface [2]. 

The work of Gillot et al. [3] showed that two out of three ACL injuries in handball are caused by non-contact situations related to technical skills and sports predispositions, such as acceleration, rotation, change in direction, jump, and braking. One of the possibilities to assess non-contact injuries was the analysis of video recordings made in 10 cases of anterior cruciate ligament rupture in women. It involves comparing the kinematics of the knee joint. This analysis showed that for all 10 injuries, the knee joint internally rotated 8 degrees in the first 40 milliseconds after contact with the ground, while an external rotation of 17 degrees was observed between 40 and 300 milliseconds. The maximum vertical ground reaction force at the moment of first contact of the foot with the ground during a change in direction or landing was three times greater than the body weight. This indicates a very dynamic development of the valgus knee joint during the first 40 milliseconds after the foot touches the ground, indicating that the valgus loading is a key factor in the mechanism of ACL damage. Furthermore, neuromuscular fatigue of the peripheral system that occurs in the macrocycle caused by the length of the season can be one of the factors that contribute to the weakening of the muscle groups surrounding the knee joint, which can change the kinematics of the knee joint and lead to more frequent valgus. It is also worth mentioning that fatigue of the muscle groups around the knee joint in women results in longer reaction times compared to men; this is visible during technical and tactical activities such as feints, acceleration, and braking, which can indirectly affect the knee joint and joint injury [3].

Among women, one of the causes that influence knee laxity may be the endocrine system and the phases of the menstrual cycle, which at the same time can be one of the internal factors that cause anterior cruciate ligament damage. A total of 6 of the 12 systematic review studies on the influence of the endocrine system on ACL rupture found increased knee laxity during the ovulatory phase of the menstrual cycle than during the follicular phase. Joint laxity did not show significant differences between the luteal and ovulatory phases and the luteal and follicular phases. Another 3 out of 12 studies similarly confirmed the highest knee laxity during the ovulatory phase of the menstrual cycle [4].

Rehabilitation of the knee joint after rupture of the anterior cruciate ligament and its reconstruction is as important as the specific nature of the surgical procedures. The improvement of the knee joint varies significantly depending on the phase of rehabilitation, and there are five phases in total. The main goal is to move from one phase to the next and, in the later appropriate stages, rebuild the strength and endurance of the knee flexors and extensors. The purpose of this is to restore the functionality of the joint from before the injury. Therefore, surgery alone may be insufficient if limb strength is not repaired [5].

The rehabilitation process after surgical reconstruction of the anterior cruciate ligament is extremely important to improve the knee joint and return to full function. Postoperative rehabilitation can also support the knee joint and reduce the risk of secondary injuries. Clinical practise guidelines for rehabilitation also define the phases of preoperative rehabilitation–prehabilitation (PR), which is the period between injury and reconstruction. The waiting time for ACL reconstruction surgery can be extended, so it is extremely important to keep the joint and the muscles surrounding the joint in high availability, which will ensure faster recovery after the procedure. There is little to moderate evidence supporting the effect of PR on functional performance before and after surgery [6]. In addition to mobilizing the patella joint, improving neuromuscular control, and improving stability and balance, the PR programme should include restoring or increasing the strength of the knee flexor and extensor muscles, including training muscular endurance, increasing muscle cross-section, and increasing neuromuscular strength [4]. In the work by Reddy et al. [7], 20 patients underwent preoperative rehabilitation, the main goal of which was to reduce pain, swelling, and inflammation. PR included strengthening exercises. In this group, there was a significant difference in the range of motion 3 and 6 weeks after surgery, but there were no differences at 3 and 6 months. The results regarding knee joint muscle strength, range of motion, and function were significantly better in patients who performed exercises before surgery than in those who did not perform PR [8].

A review of the available literature on the impact of resistance training in conditions of normobaric hypoxia revealed inconsistent research results. In the work that concerned the comparison of the impact of resistance training in conditions of normobaric hypoxia and normoxia, they found that the level of GH did not increase significantly after 6 microcycles. This is contrary to the work by [9], which states a significant increase in GH levels. This is also confirmed by Kurobe et al. [10]. They conducted a study in which they divided the participants into two groups, one trained in hypoxia and the other in normoxia, and the training included 8 microcycles with 3 units per microcycle. The group under hypoxia conditions obtained higher levels of growth hormone, but they did not go through structural changes in the muscles, although these people were able to perform a greater number of repetitions.

The clinical practise guidelines for rehabilitation also define the phases of PR, the period between injury and reconstruction. The literature is not clear on determining the duration of rehabilitation and its effectiveness in the preparation for surgery. There are scientific works that have shown positive changes in muscle structure after 6 microcycles (microcycle-7 day) of intervention [11]. Interestingly, the work [12] confirmed that PR, consisting of 5 microcycles, has positive changes in individual muscle structures. Kim et al. found that even 4 microcycles of rehabilitation can significantly reduce the strength deficit of the quadriceps femoris muscle and improve the value of jumping on one limb. It is also worth mentioning that the review [13] found negligible changes in individual muscle structures after a period of 4–6 microcycles with 2–4 training units [6].

Strength and resistance training under hypoxic conditions (hypoxia) can have positive effects in rehabilitation and pre-rehabilitation. The effect of combining training under hypoxic conditions provides favorable physiological changes in muscle tissue, indicating favorable changes in the cross-sectional area (CSA). Structural adaptations of muscles are similar under conditions of normoxia and hypoxia; however, training without adequate availability of oxygen results in an increase in the volume and cross-sectional area of muscle fibre. Proper use of hypoxia during physical exertion can increase the metabolic functions of skeletal muscle tissue. Friedmann et al. suggest that resistance training under hypoxic conditions for a period of 6 microcycles improves muscle strength and accelerates muscle growth faster than under normoxic conditions. They showed an increase in muscle endurance when performing an exercise routine under hypoxic conditions for 4 microcycles, including knee extension for 6 sets of 25 repetitions. Increased muscle endurance also had a positive effect on increasing muscle fibre CSA. This increase was greater in people training under hypoxic conditions than in people training under normoxic conditions. Furthermore, resistance training under hypoxic conditions increases the responsible mechanisms that influence hormones, cytokine, reactive oxygen species, and oxidative stress factors, which play an important role in muscle growth [14,15]. Furthermore, we were unable to find rehabilitation protocols based on a periodization scheme by taking into account the load progression parameter based on a 3:1 step load [16]. This, in our opinion, would allow precise control of the rehabilitation process in individual mesocycles with variables such as tempo and time under tension (TUT), especially in relation to hormonal changes, body composition analysis, muscle strength, and power parameters. 

Therefore, this work will focus on the impact of periodization of the PR protocol using a step load of 3;1 based on TUT and tempo on the level of muscle strength and power, as well as the concentration of GH, IGF-1, and EPO hormones under artificial high-mountain conditions. The final stage will be the impact of the protocol on VO_2_ max (maximum oxygen uptake).

A rehabilitation strength training programme before and after ACL reconstruction can be crucial in the preparation for surgery, as well as after returning to sports after ACL reconstruction. Training with an extended eccentric phase may be a better option for recovery after ACL reconstruction because it provides improved lower limb strength and improved performance in various jump tests. Stojanovic et al. [17] showed that a 6-week eccentric training programme produced a significantly greater improvement in lower limb strength than standard strength training. A longer duration of the eccentric phase has proven to be a good method of accelerating rehabilitation or PR as a result of ACL rupture due to unique neural patterns and increased mechanical muscle tension, which provides a better neuromuscular response to strength training. 

## 2. Materials and Methods

Description of the patient’s magnetic resonance imaging is as follows:

There were degenerative changes in the form of osteophytosis in the edges of the articular surfaces of the patella, femoral, and tibial condyles. The patient had Patella type II according to Wiberg. The position of the patella was correct. The SRU articular cartilages showed no changes in grade III chondropathy. The quadriceps tendon, patellar ligament, retinaculum, and the iliopsoas band of the tibia were normal. Other observations were as follows: ACL graft tear; posterior cruciate ligament with preserved continuity, with normal signal and course, positioned more vertically as in the case of ACL damage; articular cartilages of the lateral compartment with grade II chondropathy; bone marrow edema posterior to LTC; minor disruption of the inferior peripheral part of the LFC; lateral meniscus with damage to the posterior root and degenerative changes in the meniscus. The peroneal collateral ligament, the biceps femoris tendon, and the hamstring tendon were normal. In the medial compartment, the articular cartilage of the bearing surface of the MFC exhibited grade II chondropathy. Both Lakotas had the correct shape and signal, and there was damage to the medial meniscus. In the narrowed posterior horn, there was an irregular branching zone of damage and degenerative changes; the shaft was narrowed and bulged beyond the edges of the condyles. Tibial collateral ligament showed partial damage to the proximal half, grade II/II. The POL and the tendons that form the goosefoot were normal. There were features of semimembranosus tendinopathy. Hoffa’s fat body showed signs of edema. A discreetly increased amount of fluid was observed in the joint cavity, with thickening of the synovial membrane of the suprapatellar recess, as well as a collection of cystic fluid between the femur and the vastus lateralis that extends beyond the area of examination. The structure of the popliteal fossa showed no changes.

The conclusions were as follows:

- Degenerative changes.
- Chondropathy.
- Damage to the posterior horn of the MM and the posterior root of the ML.
- ACL graft tear.
- Effusion in the joint cavity.

The work uses a case study, the research material of which was a 23-year-old professional handball player. Before surgery, the patient underwent a PR protocol under normobaric hypoxia, which began 3 weeks after the injury. The patient was informed about the study protocol and its risks and benefits and then provided her written consent to participate in the study. Furthermore, the competitor had the right to resign from participating in the tests at any stage. Resignation may also occur at the request of the attending physician. The study protocol was approved by the University Bioethics Committee of the University of Physical Education; Jerzy Kukuczka in Katowice, Resolution No. 3/2021, of the University Bioethics Committee for Scientific Research of the University of Physical Education; Jerzy Kukuczka in Katowice on 21 January 2021. The problem was addressed using an experimental approach.

Research was carried out at two facilities. The intervention was carried out in the hypoxia laboratory of the University of Physical Education in Katowice. The diagnostic part took place at Galen Sp. Rehabilitation Office, Center z o. o., under the supervision of a physiotherapist and a strength and conditioning coach. Immediately after the end of the training unit, the hypoxic chamber was completely ventilated. Each trainer and device used in the rehabilitation protocol were disinfected.

Initially, 24 h before the start of the tests, the athlete was required to undergo a morphological and hormonal examination, during which the resting concentration of growth hormone (GH), insulin-like growth factor 1 (IGF-1), and erythropoietin (EPO) was measured in 6 mL of venous blood collected in the area of the antecubital fossa of the medial vein of the forearm (Table 1). Blood collection was carried out by a person with the required qualifications, observing occupational health and safety rules as follows: disposable gloves, blood collection station, skin disinfectant, multi-safe waste container, and plaster with dressing. Immediately after blood collection, it was transported to the diagnostic laboratory. Before starting the rehabilitation protocol, baseline blood counts were determined, and blood samples were collected on an empty stomach and after an overnight fast. Repeated tests were performed 30 min after the last unit of mesocycle 3 (Table 2). Furthermore, in each microcycle, 30 min after each training session, a morphological examination was performed to determine the secretion of GH according to the duration of exercise.

Hormone concentration: Growth hormone (GH) was assessed in the serum using the Beckman Coulter IV D IRMA GH Ref. IM 1397 kit (GH, (µg/L) mlU/L), using the immunoradiometric method (ImmunoTech limited liability company Praha, Czech Republic). Irisin concentration (IR, µg/mL; 0.2–2 µg/mL) was determined by the ELISA system (kit) BioVentor-Laboratorium medicina as Irisin ELISA Cat. No RAG018R, Praha Czech Republic. Insulin-like growth factor 1α (IGF-1α, ng/mL) was measured using the Beckman Coulter limited liability company Warsaw, Poland IV D IRMA IGF-1 kit Ref. A15729 ImmunoTech limited liability company Praha, Czech Republic. Erythropoietin (EPO) was measured with the Sandwich ELISA system (kit) BioVentor-Laboratorium medicina. The calibration range was 1.6–100 (mlU/mL), and the limit of detection was 0.14 (mIU/mL).

**Figure 1:**
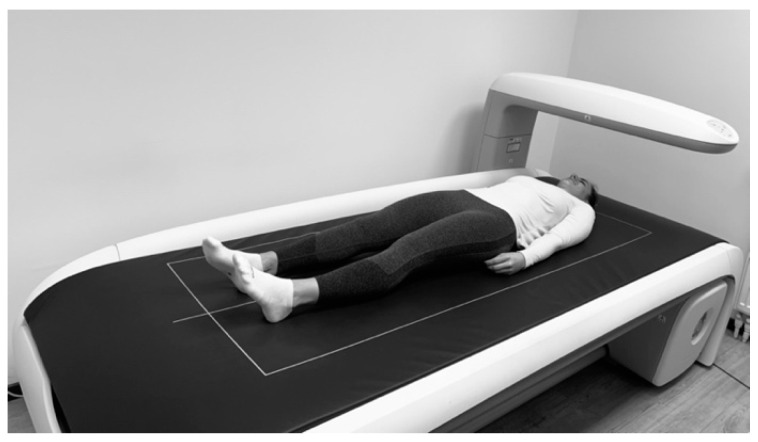
Photo during densitometry.

Before the examination, the mineral density of the examined bone was assessed by densitometry (DXA) (Figure 1). Additionally, this examination was used to analyse body composition to obtain information about the content and distribution of fat, muscles, and other tissues. Measurements were made under standard conditions, i.e., in the morning (8–9:00), 72 h before the examination and while training, and drinking alcohol or fluids containing caffeine and carbohydrates were stopped. The next measurement took place 48 h after the last 4 mesocycles.

**Figure 2:**
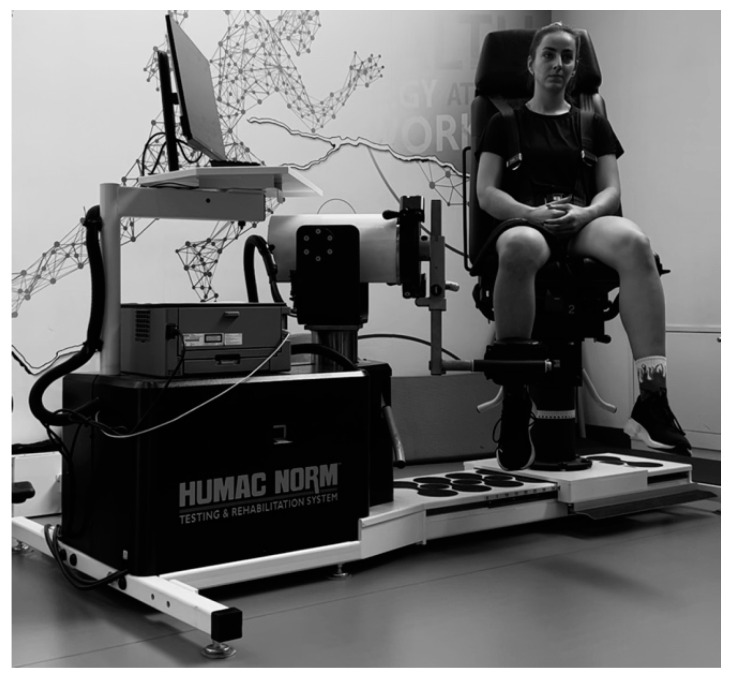
Photo during an isokinetic device test.

After consultation with the attending physician, muscle strength and maximum torque were assessed under isokinetic conditions (Figure 2). The first measurements, which were also the baseline measurements, were made 48 h before the first mesocycle, while the second measurements were made 48 h after the third mesocycle. Furthermore, the ratio of knee extensors to flexors was examined using an isokinetic device (HUMAC NORM from Boston, MA, USA). Individual muscle groups activated by concentric work (muscle contraction) under isokinetic (constant) load were evaluated under clinical conditions. The device was calibrated according to the manufacturer’s instructions. Before the test, the athlete performed a 10 min warm-up in a stationary cycle (70–80 rpm). To become familiar with the isokinetic device and the test procedure, the subject performed three submaximum and two maximum repetitions before the main test. There was a 30 s rest break between repetitions and a 3 min rest break between sets [18]. Before the test began, verbal instructions were given to generate as much force and power as possible during the test. Furthermore, no verbal prompts were used during the study, but the computer screen was set so that participants could receive feedback in real time. The subject sat in a stretched position with the backrest at an angle of 85°. The axis of rotation of the knee joint was placed in line with the axis of rotation of the dynamometer. To avoid restricting the movement of the ankle joint, a lever arm cap was attached to the head of the fibula. 

### 2.1. Knee Extensors and Flexors Were Evaluated at Angular Velocities

At −60°/s, 1 verification repetition and 5 test repetitions took place; at −120°/s, 1 verification repetition and 5 test repetitions were performed; at −180°/s, 1 verification repetition and 15 test repetitions were carried out. The range of joint mobility evaluated was determined from the extended limitation to the bending that the athlete could perform. 

### 2.2. VO_2_ Max (Maximum Oxygen Uptake) 

**Figure 3:**
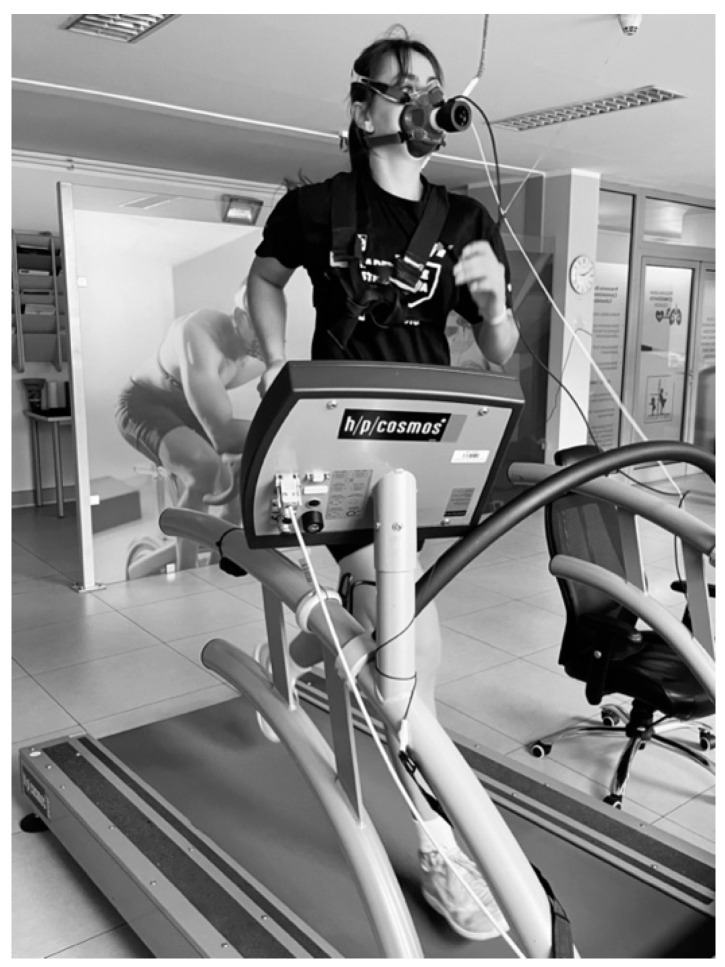
Photo during the Vo_2_max test.

Due to preparation for surgery and extensive joint damage, the maximum oxygen uptake assessment (baseline measurements) was performed after consulting a physician, and a progressive treadmill test was carried out using a modified Bruce protocol (Table 1) [19] before the start of the first mesocycle and 48 h after the last mesocycle. VO_2_ max was measured objectively and reliably in the laboratory by direct analysis of the gases involved in lung ventilation (Figure 3), and the test was carried out 48 h before the start of the first mesocycle and 48 h after the last mesocycle.

### 2.3. Resistance Training

**Figure 4:**
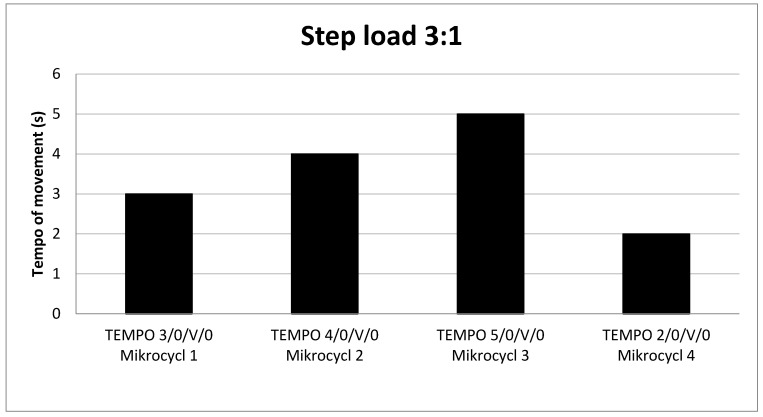
Tempo of movement (3/0/V/0) eccentric/isometric/concentric/isometric phases of each repetition/x-maximum velocity/v-volitional tempo.

The mesocycle was developed using a 3:1 progressive load progression using UNI exercises (Figure 4). The main factor that intensified the progression of the load, in addition to the nature of the exercise, was the tempo of movement (TUT–time under tension) [20,21]. The athlete tested performed three four-week mesocycles of resistance training in normobaric hypoxia at FiO_2_ = 15%, at an altitude of 3000 m above sea level, focusing mainly on the muscles of the lower extremities. Each mesocycle was divided into 4 training microcycles (Table 3) (1 microcycle = 7 days), with each microcycle containing 2 afternoon training units from 4:00 p.m. to 18:00 (Monday and Thursday) (Table 4). Each unit was preceded by a 15 min stay in the chamber, after which the competitor performed a 10 min warm-up on a bike and several resistance exercises involving the upper and lower body (5 min). The athlete then performed a set of unilateral exercises (Table 5) for the lower and upper body parts for 3 mesocycles. After strength training, endurance training was performed for 3 × 5 min run (Table 6), with a 3 min break between sets. The subject spent in the chamber from 93.5 min to 105.5 min, depending on the training microcycle (Table 7).

### 2.4. Saturation

During each training session, the subject’s saturation measurement was checked. The purpose of this measurement was to control blood oxygen saturation. Saturation was measured using a pulse oximeter. The saturation level is given as a percentage and marked with the SpO2 symbol (Table 8).

## 3. Results

### 3.1. Hormones Results

The hormone concentration determined after the last training of the last mesocycle increased compared to the test before the first mesocycle (Table 9). Especially growth hormone, which was measured after each training microcycle (Table 10). 

### 3.2. DEXA Results

Body composition analysis performed using a densitometric test showed a decrease in body weight, an increase in muscle mass, and a decrease in fat tissue 48 h after the last training microcycle, compared to the test performed 72 h before the first microcycle (Table 11). The amount of muscle mass and fat tissue in individual body segments was also specified (Table 12).

### 3.3. Isokinetic Results

#### 3.3.1. Extensors

Peak knee extensor torque in the affected and healthy leg increased significantly after completing 3 training mesocycles (Table 13).

#### 3.3.2. Extensors deficit

After 3 training mesocycles, the deficit of knee extensors between the healthy and the diseased leg decreased (Table 14).

#### 3.3.3. Flexors

Peak knee flexors torque in the diseased and healthy leg changed after completing 3 training mesocycles (Table 15).

#### 3.3.4. Flexors deficit

After 3 training mesocycles, the deficit of knee flexors between the healthy and the diseased leg decreased (Table 16).

### 3.4. VO_2_ Max Results

During the progressive treadmill test according to the modified Bruce protocol (Table 1), an increase in VO_2_ max was shown from 43.8 to 46.7 (Table 17) after completing the training protocol consisting of 3 training mesocycles.

## 4. Discussion

The test results obtained showed a 10.81 times greater increase in GH concentration in the unit preceding the deload phase. First, we believe that several factors contributed to the increased release of GH in our protocol. However, the experiment conducted showed that the main determinant that influenced the GH level in the protocol was the use of a 3:1 step load (Figure 4) based on the TUT variable (Table 2). According to [22], TUT is part of the training volume, and the rest of the training parameters in our protocol did not change and were constant. It is worth mentioning that the external load for 12 repetitions varied depending on the tempo of movement, which is why we decided to use the RIR scale in our protocol. Several studies have found that tempo combined with the number of repetitions will affect total TUT, which will consequently translate into hormonal changes [22,23,24]. It is worth referring to the work [9] where interesting results were obtained. The authors compared two different tempos: 2/0/2/0 and 5/0/3/0 for the GH level in the barbell squat exercise with 80% 1RM/5 sets/3 min breaks and repetitions until muscle failure. The highest increase in GH was achieved using a movement rate of 2/0/2/0 (13.7 ± 9.2). In turn, after the rate of 5/0/3/0, GH increased on average by (9.95 ± 7.3). When analyzing this work, it is worth referring to the average number of repetitions needed for muscle failure, which the authors recorded, and their possible impact on the level of GH in this work. The group with a faster tempo of movement (2/0/2/0) performed a significantly higher number of repetitions (59.4 ± 5.5) than the other group (42.4 ± 5.4). Another study [23] comparing GH and IGF-1 levels in three different volumes (sets) in a barbell squat at 90% 1RM with three repetitions, a tempo of 2/0/3/0, and a break of 5 min between sets showed significant changes in hormone levels. The highest concentrations were observed in 3 sets and the highest in 6 and 12 sets of barbell squat. Of course, all these studies were conducted under normoxic conditions. 

Regarding resistance training in hypoxia, it is believed that the use of low intensity and high training volume (repetitions) is the factor that can influence the level of GH in hypoxia [9,25,26,27]. Several scientific studies have shown that training with a low intensity of 50%1RM with a moderate conclusion (local hypoxia) causes an increase in GH [28,29]. On the other hand, low-intensity exercise without occlusion showed no changes in GH levels [30,31]. Another study comparing resistance training in hypoxia and normoxia consisting of two exercises, using 50% 1RM based on 5 sets of 14 repetitions, showed that increased GH secretion from the pituitary gland can be stimulated by increased metabolic accumulation, such as increased lactate and hydrogen ions in hypoxia compared to the normoxic group. Therefore, metabolic stress induced by training measures alone, such as volume and intensity, is insufficient to cause an increase in GH [9]. Unfortunately, we did not analyse the lactate level, and we can only assume, based on the above work, that it may be one of the variables that influence the GH level. However, training consisting of seven exercises, three sets of high-intensity 70% 1RM with a volume of 10 repetitions per set at FiO2 = 16.9%, showed a significant increase in GH concentration compared to the normoxic group, justifying this with increased accumulation of lactate and hydrogen ions. It should be noted that the authors found that the increase in GH was not as great as in the case of hypoxic protocols containing low intensity and high volume [14].

Another variable worth considering is the rest period between sets. Repeated sets of a high-volume resistance protocol also lead to important acute physiological responses that are mediated by rests between sets [14,32]. The use of breaks between sets in the (60–90 s) range can pose a risk of higher hormonal stress than longer periods of rest, both under normoxic and hypoxic conditions. Longer rest periods (180 s) enable the removal of intramuscular metabolites from the circulation, limiting the beneficial stimulus of metabolic stress [33,34]. To some extent, moderate or low intensity and higher volume can affect GH levels, as has been proven in several studies. It is worth comparing the method consisting of two exercises where the intensity was 50%1RM and the volume was 12 repetitions in five sets for the level of GH in hypoxia and normoxia. The break between sets and exercises was 60 s and hypoxia was established at FiO2 = 13% [25]. This study clearly states that under conditions of acute systemic hypoxia, they cause a greater response to GH levels. Although we did not investigate the influence of lactate (LA) under the conditions of the hypoxic protocol, we can only assume, based on several studies, that together with hypoxia, it is responsible for the increase in GH [31,35]. Another variable that contributes to changes in GH levels based on the literature review depends on altitude. Only a significant increase is observed from 3000 m above sea level (FiO2 = 15%), which corresponds to the height we used in our study. Additionally, GH was collected immediately after the training session [36].

In summary, we believe that the GH levels (Table 9 and Table 10) in our 3:1 step load periodization experiment were influenced by a combination of several factors. First, the higher the volume in the TUT training unit (Figure 4) with an extended eccentric phase and a large number of repetitions close to muscle failure (12-RIR-2/3), the higher the level of GH. Additionally, the use of short partial rest breaks between sets (75 s) combined with five exercises in three sets and a hypoxic environment (FiO2 = 15%) intensifies significant metabolic stress, resulting in the highest increase in GH in the main phase of step load accumulation 3:1 (Figure 4), thereby falling in the third microcycle with a total TUT of 1800 s (Table 2).

In our study, the IGF-1 level was tested 48 h before the study and 48 h after the last session. Therefore, we can only rely on the literature in assessing this parameter. We only notice a slight increase from 172 (ng/mL) to 177 (ng/mL). However, it should be noted that it is difficult to clearly recognize the physiological role of the GH/IGF-1 axis during physical exercise. It is known to cause the production of insulin-like growth factor-1 (IGF-1) from the liver, which plays an important role in skeletal muscle hypertrophy [37]. However, skeletal muscle hypertrophy can also occur without GH-stimulated increases in IGF-1 levels, possibly through other mechanisms of IGF-1 stimulation. Another aspect is the use of aerobic activities in our protocol, and based on [38], the ability to increase the concentration of circulating IGF-1 is correlated with the cardiorespiratory abilities of the subjects. It confirms our use of running units in the protocol, depending on the patient’s health capabilities, because in addition to stimulating the improvement of VO_2_ max (Table 17) in the PR process, the use of an incomplete rest break between resistance exercises in the protocol may enhance this effect.

In our protocol, we observed a significant increase in EPO from the baseline of 13.9 (mlU/mL) to 16.7 (mlU/mL) in the athlete who was tested after 24 h at the last intervention. The mechanism of hypoxia itself is quite well described. Here, the low partial pressure of oxygen causes the release of EPO, a glycoprotein that stimulates the production of red blood cells to increase oxygen carrier capacity [39,40]. Exposure to hypoxia stabilizes hypoxia-inducible factor-1α (HIF-1α) within a few minutes, resulting in transcription and production of the EPO gene. When analyzing our work, it is worth considering the duration of the intervention, both in terms of a single session and the entire periodization. Continuous exposure to hypoxia lasting between 84 and 120 min consistently increases serum EPO levels [41,42]. This would be consistent with our study because this person spent from 95.5 min to 105.5 min (Table 7), depending on the microcycle, in step load 3:1. In [43], while looking for the shortest way to stimulate the increase in EPO in intermittent hypoxia, eight cycles of 4 min intermittent hypoxia were found to have a similar effect on the EPO content as a 120 min session of continuous hypoxia. What was more interesting for us was that subsequent measurements showed that the maximum increase in EPO occurred 4.5 h after the intervention, where the group of eight cycles of intermittent hypoxia recorded an increase of 65 ± 65%, and the group of continuous hypoxia recorded an increase of 85 ± 76%. The authors concluded that eight sets of intermittent hypoxia with arterial oxygen saturation of 80% induced EPO release. Another study showed that 48 h exposure at an altitude of 2200 m above sea level increases the EPO content after 12 h (+70%) and 48 h (72%) [44]. From our point of view, monitoring this parameter may play a significant role in return to sport (RTS) protocols rather than PR, where performing RTS in hypoxia based on resistance exercises and short running sessions, especially in the final stage of rehabilitation, may accelerate the athlete’s return to full training loads.

Several factors influenced the PR periodization process under hypoxic conditions. First, the injury and associated instability of the knee joint and impaired motor control are characteristic of most ACL injuries [45]. Additionally, this injury caused cartilage and meniscus damage, which was associated with degenerative changes that also often occur with this type of injury, where chronic joint instability leads to loss of muscle strength [46]. Therefore, first of all, we used a step load of 3: 1 (Figure 4), which allowed us to avoid maladaptation and changes in critical overload, which was proven in [45]. To our knowledge, this is the first work that concerns the use of step load 3:1 in the PR protocol. Furthermore, we have not been able to find any publications that used TUT as one of the main factors in rehabilitation, combining it with hypoxia. We decided to use the tempo of movement with an emphasis on controlling the eccentric phase because it increases muscle tension. Moreover, very often the patient receives information from the trainer, such as “pay special attention to the eccentric phase” without information on how long the tempo of movement of a given motor activity should last and how it directly translates into the entire TUT. Therefore, as stated in [11], a deliberate slow tempo can be deliberately used if the load is light enough to control the structure of the movement. In our opinion, using the tempo of movement and TUT when dealing with instability can be an excellent means of periodizing the PR protocol. Hence, modifying movement tempo can indirectly cause a change in training load during a single training session, or a whole training microcycle [47,48]. The results obtained from the test on the isokinetic device of the peak torque 60 showed a significant deficit of knee extensors (24%) between the limbs, which decreased to (8%) immediately after the mesocycle (Table 14). The latest scientific reports that preoperative strength of the quadriceps muscle is related to the improvement of postoperative strength of the operated limb as well as the function of the knee joint itself and is a predictor of measuring functional results related to ACL [11]. Closed kinetic chain exercises are important for functional movement patterns that promote coactivation of the quadriceps and hamstrings and are commonly used in training to prevent ACL injuries [49]. The compressive forces experienced during weight bearing add to the stability of the knee joint, which is not experienced during open-chain kinetic exercises [50,51]. We decided to include exercises such as dumbbell step lunges and Bulgarian split squats (box 15 cm) in our mesocycle exercises, which are aimed at stimulating the quadriceps muscle in combination with an Isometric single-arm overhead lunge (Table 5). The protocol we used also influenced the maximum torque of the extensors of the injured limb (Table 13 and Table 15), where, according to many authors, the strength of the preoperative quadriceps muscle is a predictive factor of the measured functional results of ACL reconstruction [32,36]. Preoperative quadriceps strength deficit is a factor in persistent quadriceps weakness after reconstruction [52,53]. Previous studies have shown that greater muscle strength can preoperatively improve outcomes [54,55]. Therefore, several studies have suggested the addition of preoperative exercises to increase quadriceps strength prior to ACL reconstruction surgery to achieve better function after reconstruction [53,56]. The protocol we used improved muscle strength; similar results were observed in [45], which also used an increase in muscle strength and power with a 3:1 step load based on the use of variables such as tempo of movement and TUT among football players in the presession. It is stated that the rapid increase in muscle strength is due to neuromuscular and connective tissue adaptation, while the early increase in muscle CSA (cross-sectional area) size may be the result of edema. Assuming that prolonging the eccentric phase of movement may cause excessive muscle damage and delayed muscle soreness (DOMS) [45,57], we considered increasing the tempo in the eccentric phase with appropriate supercompensation during the intervention mesocycle because the change in movement of tempo was a consequence of an immediate change in TUT (tension time), which translated into training load and, consequently, a change in the total training volume affecting muscle strength [22,24,48].

However, one of the studies stated that prolonged TUT can have a negligible effect on changes in hypertrophy and muscle strength [58]. Based on the results of the densitometry test, we can determine a significant increase in muscle mass in the lower limbs (Table 13). Prolonged TUT during resistance training results in a significantly delayed stimulation of muscle protein growth after 24–30 h of recovery. Furthermore, muscle tension time increases the sharp amplitude of sarcoplasmic protein synthesis rate [59]. The results of these observations confirm that protein synthesis reactions can be influenced by extending muscle tension time and engaging as many muscle fibres as possible. Delayed stimulation of muscle protein growth is crucial along with muscle protein expression within muscle fibres, leading to an increase in the cross-sectional area of the fibre and the entire muscle [58]. 

The specific time of the muscles under tension is not clearly defined in the context of muscle hypertrophy training. According to the meta-analysis by Wilk et al. [22], TUT for a training series should last 20–70 s; in our study, the time under tension ranges between 24 and 60 s (Table 2), which seems consistent with the increase in muscle mass. In addition, TUT can depend on other factors, such as experience, type of exercise, range of motion, or external load. The research results are not clear as to the specific type of tempo, but it seems that the best solution will be to use a slow tempo in the eccentric phase and a fast tempo in the concentric phase. In our study, we used different variants of work tempo in each of the four training microcycles—3/0/V/0, 4/0/V/0, 5/0/V/0, and 2/0/V/0 (Table 2); however, in the case of the first three microcycles, the time of the eccentric phase should be noticeably longer than that of the volitional work in the concentric phase. Based on the work of Tanimoto and Ishii [60], it can be concluded that longer TUT in resistance training may contribute to increased hypertrophy according to the cross-sectional area of the muscle. They compared three training programmes, but two of them took into account the same external load, 50%RM, which differed in the work tempo: medium 3/1/3/0 and fast 1/1/1/0. The subjects performed three sets of knee extensions until muscle fatigue, with 60 s breaks, for a period of 12 weeks. When comparing these two training programmes with the same external load, the results clearly show that medium-tempo training, 3/1/3/0, was more effective for hypertrophy than fast-tempo training, 1/1/1/0. Therefore, it can be concluded that in the case of these two training programmes, it was TUT that was decisive. A longer TUT may be important for muscle growth because it causes greater metabolic stress and hormonal reactions, which are some of the factors that influence muscle hypertrophy, which indirectly affects strength [59]. Kurobe et al. [10] found an increase in the triceps brachii muscle in the group of subjects trained under norbaric hypoxia than in the normoxia group. The increase in muscle thickness was associated with a higher concentration of GH after exercise, which was also observed in training in hypoxia. GH leads to an increase in the concentration of IGF-1, which is crucial in the hypertrophic adaptation of muscles. The results of Kurobe et al. [10] work are similar to the results of our work, in which we also confirm an increase in GH, IGF-1 (Table 9), and an increase in lean body mass (Table 11). The effect of hypoxia on muscle hypertrophy is not fully known, but it is certain that the number of metabolites accumulated during low- or moderate-intensity exercise is higher than during training in normoxia. Increased accumulation of metabolites can play a key role in muscle mass growth [32]. Guardado et al. [61] achieved similar results to ours in their work. He compared two subjects, one of them trained in norbaric hypoxia and the other in normoxia. The training programme was carried out over a period of 7 weeks. Based on two measurements, before the start of training programmes and after a 3-week detraining period, the results were clear regarding the benefits of training under hypoxic conditions. Group training under hypoxia conditions achieved a greater increase in muscle mass and a greater decrease in fat mass. Based on this study, it can be concluded that increasing muscle mass and reducing fat tissue, in hypoxic conditions, can be concluded.

## 5. Conclusions

The periodization of PR in hypoxia based on a case study shows that increasing the eccentric phase increases the level of GH in hypoxia several times. Combining this aspect with the role of GH in the body, which helps cells and tissues grow and regenerate, refers to [60] in which it was found that GH treatment after ACL reconstructive surgery can prevent the loss of strength and muscle mass of the injured limb. Using a hypoxic environment at an appropriate altitude combined with changes such as a short rest break between sets and controlling the tempo of movement with an eccentric phase, TUT, can be beneficial. The use of a step load with a deload phase allows the occurrence of supercompensation, which may be crucial in the reliable and repeatable diagnosis of an injured athlete in the rehabilitation process. Additionally, step load allows one to avoid critical overload changes related to rehab during the return to sport (RTS) process. Therefore, we believe that it is necessary to extend this work to a larger number of participants who implement this protocol in hypoxia and normoxia, and extending the research to lactate levels would increase the value of the work. In summary, we believe that this protocol can be an alternative to the PR process, especially among athletes who care about fast RTS.

## Figures and Tables

**Table 1:** Progressive exercise test.

STAGE	SPEED (km/h)	Elevation (%)	Duration (min)
1	6	0	3
2	8	0	3
3	10	0	3
4	12	2.5	3
5	12	5	3
6	12	7.5	3
7	12	10	3
8	12	12.5	3
9	12	15	3

**Table 2:** Tempo of the microcycles.

TIME UNDER TENSION (S)					
MICROCYCLE TEMPO	TUT Exercise (s)			Total TUT per Training Session (s)	Total TUT per Training Session (min)
	TUT per Limb in a Series	TUT on Both Limbs in a Series	Total TUT in the Exercise		
3/0/V/0	36	72	216	1080	18
4/0/V/0	48	96	288	1440	24
5/0/V/0	60	120	360	1800	30
2/0/V/0	24	48	144	720	12

**Table 3:** Resistance training variables.

Variables	Unilateral
RIR (Reps in reserve)	2–3
Sets (n)	3
Rest Between Sets (s)	75
Rest Between Exercises (s)	180
Reps (n)	12 (12 per side)
Number of Exercises (n)	5

**Table 4:** Microcycle variables.

Microcycle	1		2		3		4	
**Training days**	M 4–6 PM	U+ 3 × 5′R	M 4–6 PM	U+ 3 × 5′R	M 4–6 PM	U+ 3 × 5′R	M 4–6 PM	U+ 3 × 5′R
	T 4–6 PM	U+ 3 × 5′R	T 4–6 PM	U+ 3 × 5′R	T 4–6 PM	U+ 3 × 5′R	T 4–6 PM	U+ 3 × 5′R

M—Monday; T—Thursday; U—Unilateral; R—Running.

**Table 5:** Exercise type.

Numbers	Unilateral Exercise in 1, 2, 3 Mesocycle
1.	Dumbbell step lunges: Standing position with dumbbells in hands, taking a lunge forward, maintaining the center of gravity between the legs, and then lowering the position and returning to the standing position; changing legs and repeating the same movement.
2.	Sandbag single-leg deadlift: Single-leg standing position with a sandbag placed on the back, maximum flexion of the hip joint, wrapping the torso and lifting the free leg with the weight, and returning to the single-leg standing position.
3.	Bulgarian split squat (box 15 cm): One-legged standing position, with opposite leg placed on a 15-cm box; performing a squat on the standing leg, optionally with an external weight in the hands, and returning to the standing position with one leg.
4.	Eccentric isometrics one leg glute bridge: Performing a hip raise on two benches, with the back placed on one of the benches and the exercising leg on the other; flexion of the hip joint below the height of the benches, followed by extension.
5.	Isometric lunge with shoulder overhead press: Performing an isometric lunge squat, with the knee raised above the ground while pressing dumbbells above the head.

**Table 6:** Running session.

Mesocycle	1	2	3
Training	3 × 5′R/180 s B Tempo-6:00–5:30 min/km	3 × 5′R/150 s B Tempo-5:45–5:15 min/km	3 × 5′R/150 s B Tempo-5:15–5:00 min/km

R—running, B—break between running set.

**Table 7:** Time in hypoxia.

Action	Duration/Time of Training in Hypoxia (min)			
	1 Microcycle	2 Microcycle	3 Microcycle	4 Microcycle
Passive and Staying Passive	15	15	15	15
Warm-up	15	15	15	15
Running Set	15 (3 × 5′)	15 (3 × 5′)	15 (3 × 5′)	15 (3 × 5′)
Break between running set	9 (3 × 3′)	9 (3 × 3′)	9 (3 × 3′)	9 (3 × 3′)
Exercise time	18 (TUT)	24 (TUT)	30 (TUT)	12 (TUT)
Break between exercises	10 (5 × 2′)	10 (5 × 2′)	10 (5 × 2′)	10 (5 × 2′)
Break between sets	12.5 (10 × 75″)	12.5 (10 × 75″)	12.5 (10 × 75″)	12.5 (10 × 75″)
Staying passive	5	5	5	5
**Total time in hypoxia**	99.5	105.5	110.5	93.5

**Table 8:** Saturation measurements.

Measurement	SpO2 (% +/−)
Before warm-up	95–91
After warm-up	90–84
After each exercise	85–79

**Table 9:** Blood test results.

Variables	Before 1st Mesocycle	After 3st Mesocycle
GH—growth hormone	0.58 (ng/mL)	6.27 (ng/mL)
EPO—erythropoietin	13.9 (mlU/mL)	16.7 (mlU/mL)
IGF-1—insulin growth factor	172 (ng/mL)	177 (ng/mL)

**Table 10:** Growth hormone results.

GH-Growth Hormone			
TEMPO	TUT (s)	ng/mL	Progress
Before		0.58	100
3/0/2/0	120	4.9	8.4
4/0/2/0	144	5.4	9.31
5/0/2/0	168	6.27	10.81
2/0/2/0	96	4.82	8.31

**Table 11:** Body analysis.

Analysis	Before	After
High (cm)	180	180 (cm)
Weight (kg)	73	71.7 (kg)
Bone mineral content (kg)	3.18	3.18 (kg)
Bone mineral density (g/cm^3^)	1377	1377 (g/cm^3^)
Soft tissue (kg)	69.99	68.02 (kg)
Fat tissue (kg)	21.29	16.96 (kg)
Muscle mass (kg)	48.70	51.56 (kg)

**Table 12:** Segment analysis.

Region	Fat Mass (%)		Total Mass (kg)		Fat Mass (g)		Muscle Mass (g)		Bone Mineral Content (g)	
	Before	After	Before	After	Before	After	Before	After	Before	After
Right arm	26.1	25.8	4.3	4.4	1354	1027	2742	3147	204	204
Left arm	26.1	25.8	4.3	4.4	1354	1027	2742	3147	204	204
Damaged limb	32.3	30.8	13.9	14.6	4745	4174	8553	9841	602	602
Healthy limb	30.1	29.5	14.5	14.7	4699	4087	9148	10,031	653	653
Torso	26.1	25.2	31.3	30.6	8280	7482	22,031	22,158	989	989

**Table 13:** Peak torque extensors.

Variables	Damaged Limb			Healthy Limb		
	Before	After	Growth (%)	Before	After	Growth (%)
Peak torque 60′/s-(Nm/FFM)	159	183	13%	209	199	−5%
Peak torque 120′/s-(Nm/FFM)	145	159	9%	165	160	−3%
Peak Torque 180′/s-(Nm/FFM)	121	125	3%	130	128	−2%

**Table 14:** Deficit in extensors.

Variables	Deficit (%)	
	Before	After
Peak torque 60′/s-(Nm/FFM)	24	8
Peak torque 120′/s-(Nm/FFM)	12	1
Peak Torque 180′/s-(Nm/FFM)	7	2

**Table 15:** Peak torque flexors.

Variables	Damaged Limb			Healthy Limb		
	Before	After	Growth (%)	Before	After	Growth (%)
Peak torque 60′/s-(Nm/FFM)	113	116	3%	115	118	3%
Peak torque 120′/s-(Nm/FFM)	91	98	7%	92	102	10%
Peak Torque 180′/s-(Nm/FFM)	80	83	4%	84	89	6%

**Table 16:** Deficit in flexors.

Variables	Deficit (%)	
	Before	After
Peak torque 60′/s-(Nm/FFM)	2	2
Peak torque 120′/s-(Nm/FFM)	1	4
Peak Torque 180′/s-(Nm/FFM)	5	6

**Table 17:** Vo_2_max.

Variables	Before	After
**FINAL LOAD (km/h)**	12 < 5	12 < 7.5
**FINAL LOAD FINAL LT (km/h)**	10	12
**VO_2_MAX (L/′)**	3.1	3.17
**VO_2_MAX (mL/kg/′)**	43.8	46.7
**VO_2_ LT (mL/kg/′)**	33.9	38
**VEmax (L/′)**	117.5	117.7
**RERmax (VCO_2_/VO_2_)**	1.19	1.14
**HRmax (ud/′)**	197	198
**HR LT (ud/′)**	177	183
**LAmax (mmol/L)**	8.96	9.9
**LA 12**′ **res (mmol/L)**	−3.04	−1.56

HRmax: maximal heart rate; VEmax: maximal ventilation; RER—respiratory exchange ratio; LA—lactate.

## Data Availability

The datasets generated and analyzed during the current study are not publicly available but are available from the corresponding author who organized of the study.
